# May-Thurner variant secondary to degenerative lumbar spondylolisthesis: a case report

**DOI:** 10.1259/bjrcr.20170011

**Published:** 2017-08-04

**Authors:** David McKean, James Allman Sutcliffe, Hassan El Hassan, Nassim Parvizi, Anuj Wali, Dinuke Warakaulle, James Teh, Edward Seel, Stuart Blagg, Richard J Hughes

**Affiliations:** ^1^Radiology Department, Stoke Mandeville Hospital, Buckinghamshire Healthcare NHS Trust, Aylesbury, UK; ^2^Radiology Department, Oxford University Hospitals NHS Trust, Headington, Oxford, UK; ^3^Orthopaedic Department, Stoke Mandeville Hospital, Buckinghamshire Healthcare NHS Trust, Aylesbury, UK

## Abstract

May-Thurner syndrome (MTS) is a rare condition in which patients develop iliofemoral deep venous thrombosis due to an anatomical variant in which the right common iliac artery overlies and compresses the left common iliac vein against the lumbar spine. We report a case of variant MTS, where vascular distortion secondary to spontaneous spinal arthrodesis of degenerative lumbar spondylolisthesis resulted in left common iliac vein compression and iliofemoral deep vein thrombosis. While the common complications of degenerative spondylolisthesis, such as spinal stenosis, are well described; the potential for pelvic vascular distortion secondary to anterior translation of the lumbar spine is not well recognized. The purpose in presenting this case is to describe the mechanism by which this variant of MTS occurs and highlight the need for vigilance for this unusual clinical entity.

## Case report

A 64-year-old female patient with background of a right iliac fossa renal transplant presented to the renal outpatient clinic complaining of unilateral swelling of the left lower limb for several weeks. The clinical history did not reveal any risk factors for deep vein thrombosis. On clinical examination the patient had unilateral leg swelling on the left with pedal oedema. Observations were normal and cardiovascular and abdominal system examinations were unremarkable.

## Investigations

Biochemical investigation of the patient’s renal function revealed normal values.

**Figure 1.**
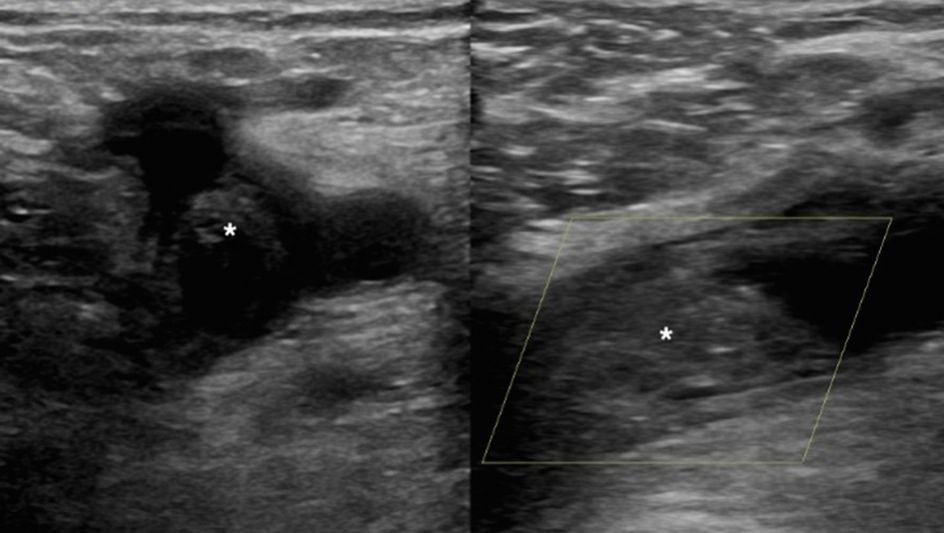
Transverse and longitudinal images of the common femoral vein demonstrating echogenic thrombus (white asterisks).

Bilateral lower limb venous Doppler ultrasonography (US) revealed extensive thrombus within the left popliteal vein, superficial femoral vein with antegrade extension into the left common femoral vein (Figure 1). The patient promptly underwent CT to exclude occult malignancy.

**Figure 2.**
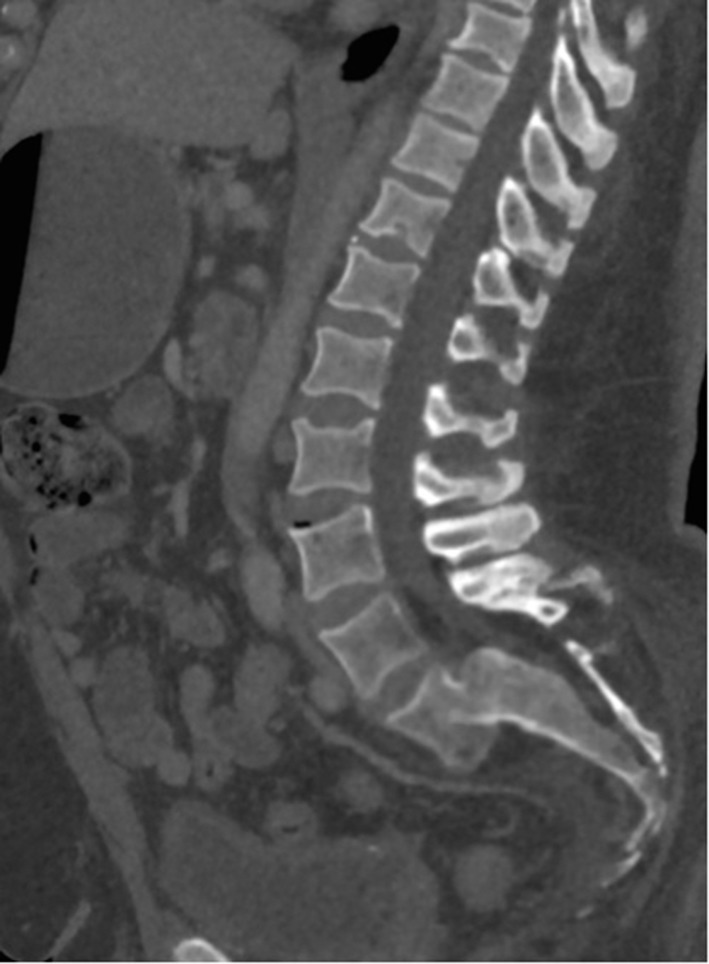
Midline sagittal reconstruction CT demonstrating Grade III degenerative spondylolisthesis of the L5 vertebral body on the S1 vertebral body with evidence of spontaneous arthrodesis.

**Figure 3.**
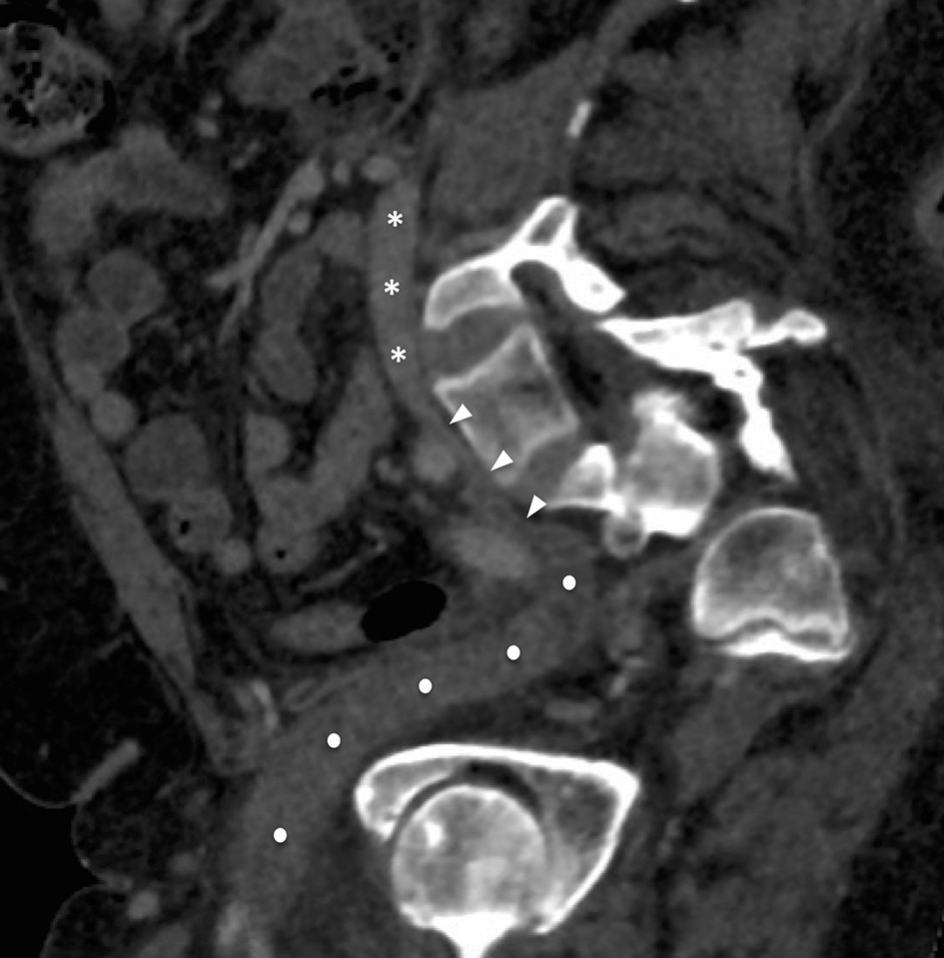
Midline sagittal oblique reconstruction demonstrating variant May-Thurner syndrome secondary to lumbar degenerative spondylolisthesis. There is extensive thrombus within the expanded left external iliac vein (white circles). There is variant May-Thurner Syndrome with compression of the left common iliac vein secondary to anterior translation of the lumbar spine (white arrow heads). The course of the inferior vena cava is indicated by the white asterisks.

**Figure 4.**
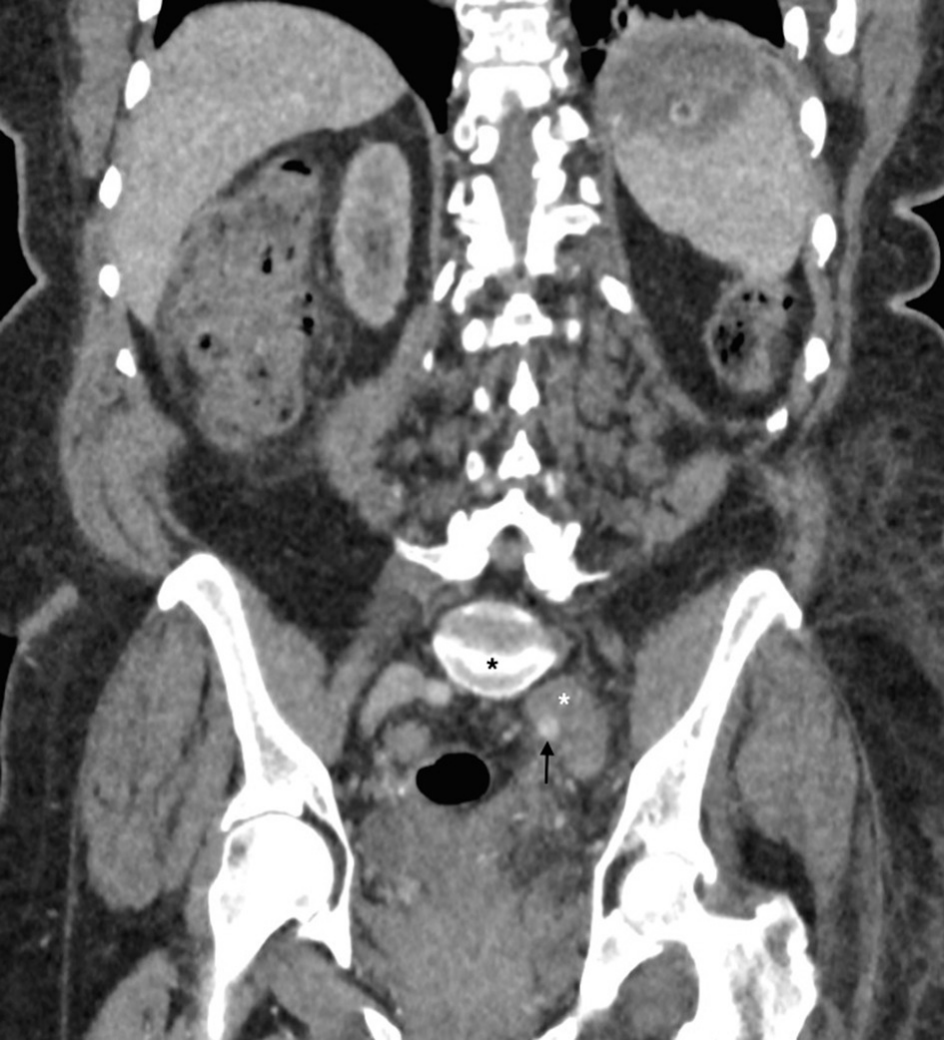
Coronal reconstruction CT demonstrating compression of the left common iliac vein between the right common iliac artery (black arrow) and anteriorly translated lumbar spine (black asterisk). Low attenuation thrombus is seen within the expended left external iliac vein (white asterisk). Note left-sided soft tissue oedema secondary to deep venous thrombosis.

CT demonstrated Grade III degenerative spondylolisthesis of L5 on S1 and spontaneous spinal arthrodesis at this level (Figure 2). Anterolisthesis of the L5 vertebral body resulted in anterior displacement and distraction of the common iliac vein. This resulted in compression of the left common iliac vein by the overlying right common iliac artery and left internal iliac artery. In contrast to classical May-Thurner syndrome (MTS), where a venous spur occurs at the level of compression by the right common iliac artery, in this case the length of venous compression is more extensive extending caudally to the inferior limit of the spondylolisthes (Figure 3). Extensive low-attenuation thrombus was evident within the expanded left internal iliac vein with retrograde extension into the left superficial femoral and popliteal veins (Figures 3 and 4). Subsequent haematological tests excluded hypercoagulability or thrombophilia as the cause for the deep venous thrombosis (DVT).

## Outcome and follow up

The case was discussed at our Orthopaedic and Vascular multidisciplinary team meetings (MDTs). The patient recovered well following a prolonged course of anticoagulation therapy with follow-up arranged to discuss with the patient the possibility of venoplasty and subsequent venous stenting. There has been no recurrence of DVT in the ensuing 6 months.

## Discussion

Degenerative spondylolisthesis is a common condition whereby chronic instability and intersegmental degenerative changes result in the slip of one vertebral body over the one below with a reported prevalence of up to 13.6% in the general population.^1^

While the potential complications of degenerative spondylolisthesis with regards to the development of low back pain and spinal stenosis are a focus of ongoing research and debate,^2,3^ complications secondary to distortion of the pelvic organs or vasculature are not well described. We report a case of iliofemoral DVT secondary to lower lumbar spondylolisthesis with compression of the left internal iliac vein between the anteriorly displaced L5 vertebral body and overriding right common iliac artery. This case highlights the need for a high index of suspicion for this variant of MTS secondary to spondylolisthesis as prompt diagnosis and appropriate management can help prevent long-term complications.

May and Thurner originally described compression of the left common iliac vein in 1957^4^ when they reported thickening of the left common iliac vein at the location where it was crossed and compressed against the fifth lumbar vertebrae by the overlying right common iliac artery. The prevalence of MTS has been reported to be 22–24% in a retrospective analysis of CT scans.^5^ However the clinical prevalence of DVT related to MTS is low, with iliac vein compression syndrome occurring in only 2 to 3% of all lower extremity DVTs.^6^ Patients with MTS typically present with acute unilateral left lower limb swelling and pain. Complications of chronic MTS may include skin pigmentation changes, varicose veins, chronic leg pain and recurrent skin ulcers.

While numerous case reports have described the presence of an over-riding right common iliac artery resulting in MTS, this case highlights the unusual variant of lumbar spondylolisthesis resulting in anterior translation of the lumbar spine, left common iliac vein compression and DVT.

Spondylolisthesis is displacement of a vertebral body on the one below and may result from several etiologies, of which the most common are spondylolysis and spondylotic degeneration.^7–9^ In our patient chronic degenerative spondylolisthesis at L5/S1 had resulted in spontaneous spinal arthrodesis, which may occur to some extent in as many as 20.9% of patients with spondylolisthesis. While the complications of spinal segmental instability with regards to neurological complications and back pain are well described,^10–12^ the potential for distortion of the pelvic vasculature has not previously been widely recognized. In light of this clinicians must have a high degree of vigilance for MTS in the context of known spondylolisthesis.

The anatomical defect associated with MTS occurs high in the pelvis, an area that is not easily visualized by ultrasound.^13^ As such, if MTS is suspected, CT venography or magnetic resonance venography should be performed with a view to early commencement of appropriate therapy. Definitive therapy of symptomatic MTS requires a multidisciplinary approach. A variety of surgical treatment options have been reported. These have included vein-patch angioplasty with excision of the intraluminal bands, division and relocation of the right common iliac artery behind the left common iliac vein or inferior vena cava, or contralateral saphenous vein graft bypass to the ipsilateral common femoral vein. Long-term patency of these surgical options has been reported as 40–88%.^14^ In recent years, treatment with endovascular techniques has been described, namely venoplasty and stenting.^14,15^ The long-term results are very encouraging with primary, assisted primary and secondary patency rates of 79%, 100% and 100% at 6 years in patients with primary iliac vein obstruction.^16^ The risk of complication following endovenous recanalization is low with mortality ranging from 0% to 1% and the most common complications, early and late thrombosis, occurring in 1.5–3% and approximately 5% of cases, respectively.^17^

In conclusion, distortion of the pelvic vasculature is a potential complication of lumbar spondylolisthesis that may result in variant MTS Musculoskeletal radiologists, who may often be responsible for reporting cross sectional imaging of the lumbar spine, must be vigilant for filling defects within the pelvic vessels or abnormal expansion of the left common iliac vein in the context of spondylolisthesis as early diagnosis and appropriate management of this condition are essential to help prevent the long-term complications of chronic venous obstruction.

## Learning points

1. The case illustrates the risk of distortion of the pelvic vasculature secondary to lumbar spondylolisthesis.
2. In the event of such vasculature distortion there is a risk of variant May-Thurner syndrome whereby the left common iliac vein is compressed by the anteriorly translated lumbar spine. This may result in left-sided venous thrombosis.
3. Radiologists should have a high index of suspicion for such variant causes of May-Thurner syndrome in the context of spondylolisthesis and such cases should be discussed at the local MDT as venoplasty or venous stenting may be required to treat these patients.

## Consent

Written informed consent for the case to be published (including images, case history and data) was obtained from the patient(s) for publication of this case report, including accompanying images.
